# Precision Bariatric/Metabolic Medicine and Surgery

**DOI:** 10.3390/jcm12051909

**Published:** 2023-02-28

**Authors:** Laurent Genser, Dominique Thabut, Judith Aron-Wisnewsky

**Affiliations:** 1 Sorbonne Université, Assistance Publique-Hôpitaux de Paris, AP-HP, Department of Hepato-Biliary and Pancreatic Surgery, Pitié-Salpêtrière University Hospital, 47-83 boulevard de l’Hôpital, 75013 Paris, France; 2 Sorbonne Université, INSERM, Nutrition and Obesity: Systemic Approaches, NutriOmics, 75013 Paris, France; 3 Sorbonne Université, Assistance Publique-Hôpitaux de Paris, AP-HP, Department of Hepato-Gastroenterology, Pitié-Salpêtrière University Hospital, 47-83 Boulevard de l’Hôpital, 75013 Paris, France; 4 Sorbonne Université, Assistance Publique-Hôpitaux de Paris, AP-HP, Department of Nutrition, Pitié-Salpêtrière University Hospital, 47-83 Boulevard de l’Hôpital, 75013 Paris, France

Indications and techniques of bariatric surgery (BS) have constantly evolved in recent decades and now face new challenges. 

BS has demonstrated the ability to induce durable weight loss, improve metabolic alterations, prevent metabolic disorders, and be used for the long-term management of obesity as opposed to intensive medical treatment [1]. Furthermore, BS reduces cancer incidence as well as cardiovascular risks in patients with obesity, causing a 5-year increase in life expectancy [2,3]. Specifically, in patients with obesity and T2D, randomized clinical trials [4,5,6,7,8,9,10,11,12,13,14,15] (RCT) and long-term observational controlled studies [16,17] examining the efficacy of surgical treatment versus intensive medical care have all verified the effectiveness of surgery. This is the case for both the control of glycemic control (T2D remission is obtained in 50% of patients) [16] and improvement in microvascular complications [17], such as diabetic nephropathy [18] and diabetic retinopathy [19]. In addition, BS increases life expectancy by 9 years in patients with T2D [2]. Altogether these data create a paradigm shift with the proposed inclusion of bariatric procedures within the treatment algorithm of T2D [20]. France has recently recommended metabolic surgery to be performed in patients with grade I obesity and uncontrolled T2D [21].

The safety profile of BS has constantly improved in recent decades. The implementation of the centralized management of bariatric surgery complications, concomitant with the maturation of the endoscopic management of bariatric-related complications (i.e., stenosis, leaks) [22,23] in expert centers, led to a significant decrease in the postoperative mortality following bariatric procedures [24]. Data from Benchmark studies confirmed that BS-related mortality was almost nil settings in expert centers, both in primary and revisional settings [25,26]. Such data should warrant a wide adoption of BS integrated into multidisciplinary teams and reassure patients with T2D, as well as diabetologist specialists, of these operations, performed in the case of poor glycemic control despite optimal medical management.

Although invasive, BS remains the most effective therapy for achieving sustainable weight loss. Recently pharmacological treatments for obesity have emerged, providing an alternative to surgery in the case of limited lifestyle intervention efficacy. Glucagon-like Peptide-1 (GLP-1) receptor analogues demonstrated significant dose-dependent weight loss outcomes (mean 15% weight loss) concomitant with improved comorbid condition and moderate side effects (mostly limited to gastro-intestinal disorders) [27]. As expected (obesity being a chronic disease), treatment cessation led to significant weight regain one year after interruption, suggesting either long-term treatment maintenance or the need to add synergic therapeutics (e.g., surgery) [28]. Importantly, other medical treatments such as double GIP (gastric inhibitory polypeptide)/GLP-1 analogs cause a more significant dose-dependent weight loss (mean 22% weight loss), and this treatment will most likely become available in the future [29]. 

With the maturation of the endoscopic sleeve gastroplasty (ESG), the endoscopic management of obesity is also becoming a possibility. In the MERIT randomized controlled study [30], patients undergoing ESG experienced greater weight loss than under lifestyle intervention alone (% total weight loss: 13.6% versus 0.8% at two years) with only a minor reported morbidity. However, the long-term sustainability and low-morbidity profile of this management remains to be reported and validated by other RCTs to warrant the widespread and safe use of ESG. Altogether, these outcomes are encouraging and may potentially enlarge the therapeutic arsenal for the management of obesity and its related diseases, evolving toward more personalized metabolic multidisciplinary management.

Finally, as well as technical considerations, bariatricians should also rethink bariatric patient care pathways and, more specifically, postoperative follow-up to warrant patient’s adhesion, and thus the long-term metabolic benefits of maintenance and safety. Patients lost to follow-up after BS are at risk of poorer weight loss [31] and increased morbidity (specifically with bariatric-related complications) [32]. Less than 50% of patients operated on using BS are still in active follow-up, even in expert centers five years after a bariatric procedure [31,33], demonstrating the potential of improving such a relevant clinical indicator. The recent COVID-19 pandemic showed us that bariatric teleclinics were a satisfactory and safe substitute to conventional clinics, providing maintained bariatric follow-up during, and beyond the pandemic, with a 93.5% follow-up rate at two years from implementation [34]. Nevertheless, other discoveries remain to be made. 

This Special Issue aims to highlight recent data on the outcomes, risks prognosis and development trends in previously unexplored aspects of obesity management.
